# Zika, contraception and the non‐identity problem

**DOI:** 10.1111/dewb.12176

**Published:** 2017-11-12

**Authors:** Keyur Doolabh, Lucius Caviola, Julian Savulescu, Michael Selgelid, Dominic JC Wilkinson

**Keywords:** bioethics, empirical ethics, ethics, health priorities, resource allocation

## Abstract

The 2016 outbreak of the Zika arbovirus was associated with large numbers of cases of the newly‐recognised Congenital Zika Syndrome (CZS). This novel teratogenic epidemic raises significant ethical and practical issues. Many of these arise from strategies used to avoid cases of CZS, with contraception in particular being one proposed strategy that is atypical in epidemic control.

Using contraception to reduce the burden of CZS has an ethical complication: interventions that impact the timing of conception alter which people will exist in the future. This so‐called ‘non‐identity problem’ potentially has significant social justice implications for evaluating contraception, that may affect our prioritisation of interventions to tackle Zika.

This paper combines ethical analysis of the non‐identity problem with empirical data from a novel survey about the general public's moral intuitions. The ethical analysis examines different perspectives on the non‐identity problem, and their implications for using contraception in response to Zika. The empirical section reports the results of an online survey of 93 members of the US general public exploring their intuitions about the non‐identity problem in the context of the Zika epidemic. Respondents indicated a general preference for a person‐affecting intervention (mosquito control) over an impersonal intervention (contraception). However, their responses did not appear to be strongly influenced by the non‐identity problem.

Despite its potential philosophical significance, we conclude from both theoretical considerations and analysis of the attitudes of the community that the non‐identity problem should not affect how we prioritise contraception relative to other interventions to avoid CZS.

## 1 INTRODUCTION

Zika virus attracted global attention in November 2015 when it was declared a public health emergency by Brazil, and shortly thereafter a Public Health Emergency of International Concern by the World Health Organization.^1^ While there was only limited understanding of the virus at the time of these declarations, the outbreak of infection in Brazil coincided with a surge in reported cases of severe fetal microcephaly, a condition associated with severe physical and intellectual disability later in life. Since that time, researchers have established that intrauterine Zika infection is the cause of a group of symptoms collectively called Congenital Zika Syndrome (CZS) that often, but not always, includes microcephaly.^2^^,^ 3 This is the first novel teratogenic disease in over 30 years.^4^

Zika is an arbovirus, spread primarily by *Aedes* mosquitos and to a lesser degree by mosquitoes from other genera such as *Culex*.^5^^,^ 6 Many of the interventions that have been used in response to Zika are familiar from other mosquito‐borne diseases. These measures include spraying insecticide, removing mosquito breeding sites and personal protection from mosquitos with repellent and appropriate clothing. However, one of the interventions suggested to reduce the incidence of CZS is less usual in epidemic control: contraception. Contraception can prevent CZS by delaying pregnancy until the risk of Zika infection is lower – either until the peak season of Zika transmission has passed, or until the outbreak has been controlled. Brazil, Colombia, Jamaica, Ecuador and El Salvador all advised that women delay their pregnancy for up to two years to reduce the threat of CZS.^7^

There are some long‐standing ethical objections to the use of contraception.^8^ Religious objections are probably the most familiar, and are usually based on concerns that contraception is unnatural, could increase promiscuity and extra‐marital sex, and may be considered a form of abortion (particularly in the case of abortifacients, which prevent fertilized ova from implanting into the uterus). There are also safety concerns, especially regarding hormonal contraception, which can have side effects and may not reduce the transmission rates of sexually transmitted infections.^9^ To maximise uptake, contraception must be combined with sexual health education, and implemented effectively.^10^ Some have expressed concerns that the burden of contraception, and particularly hormonal contraception, inequitably falls upon women.^11^ These ethical concerns might explain why less funding has been made available for providing contraception to at‐risk women than for research and mosquito control, despite it being a potentially effective intervention; of the USD$1.1 billion of aid that the US government provided to combat Zika, none was allocated to increasing contraception availability.^12^

However, using contraception to reduce the burden of CZS has an additional ethical complication. Interventions that have an impact on the timing of conception are qualitatively different from other public health interventions because they potentially alter which people will exist in the future. The philosopher Derek Parfit first called this effect ‘the non‐identity problem’ in his 1984 book *Reasons and Persons*,^13^ which included thought experiments that closely mirror current questions relating to Zika virus.

The non‐identity problem has been said to have major implications for reproductive ethics.^14^ For example, it has been said to render the “best interests of the child” standard (which is the foundation stone of legislation on assisted reproduction) as irrelevant. Jan Narveson has famously argued that the right solution to the non‐identity problem is to adopt a person‐affecting ethic.^15^ We will describe this. Applied to the debate over response to Zika, a person‐affecting ethic would make contraception a low or no priority intervention.

The non‐identity problem may have other major implications for health policy and social justice. Social justice is concerned with the wellbeing of the population, but controversy surrounds questions about how to balance the interests of current people against the interests of future or possible people. Some philosophers have argued that the non‐identity problem has crucial implications for physicians’ moral duties.^16^ Non‐identity is also thought to threaten the case for intergenerational compensation of past injustices.^17^ Whether or not public health interventions are identity‐affecting might thus affect the priority that we ought to give to them. This could have implications not only for our approach to Zika, but to other epidemics of teratogenic diseases like rubella, toxoplasmosis and parvovirus.^18^^,^ 19 ^,^ 20

In this paper, we combine ethical analysis of the non‐identity problem with empirical data about the general public's moral intuitions. The ethical analysis will describe different responses to the non‐identity problem and their implications for using contraception in response to Zika. The empirical section will describe a survey of the US general public, exploring their intuitions about the non‐identity problem in the context of the Zika epidemic. We conclude, based both on our normative analysis and the intuitions of ordinary people, that the non‐identity problem should not affect how we prioritise contraception relative to other public health interventions against Zika and CZS.

## 2 ETHICAL ANALYSIS

### 2.1 CZS and non‐identity

Current methods of responding to Zika are fairly limited. Because there are no treatments or vaccines (although both are under development),^21^^,^ 22 contraception is one of the few potentially effective interventions available to prevent CZS besides mosquito control methods. Contraception's efficacy relates to the fact that fetuses are most at risk of developing CZS if their mothers are infected during the first trimester of pregnancy. To avoid the birth of an affected infant, women could delay becoming pregnant until the peak season of *Aedes* mosquitos has passed – the seasonality of Zika has not yet been established, but according to some estimates delaying pregnancies by a few months could reduce the CZS risk of 88,000 pregnancies in Brazil.^23^ Alternatively, women could delay their pregnancies until other interventions have reduced the transmission rates of Zika, or else until the disease has been cleared from the area entirely. Barrier methods of contraception may also reduce the chance of sexual spread of the disease.^24^

As noted, using contraception to avoid CZS potentially changes which people will exist in the future. If a woman waits a month to conceive, she will have produced a different ovum for fertilisation, and the subsequent child will be genetically different from the one who would have been born if she had not delayed her pregnancy. If we think that genetic makeup is a necessary part of an individual's identity, contraception will therefore change who exists. Moreover, a much shorter delay may still be sufficient to change identity if it changes which of the millions of possible spermatozoa ends up fertilising the ovum. This identity effect would certainly apply in the case of Zika, since the aim is to delay pregnancy for at least the duration of the peak season of *Aedes* mosquito activity.

**Figure 1:**
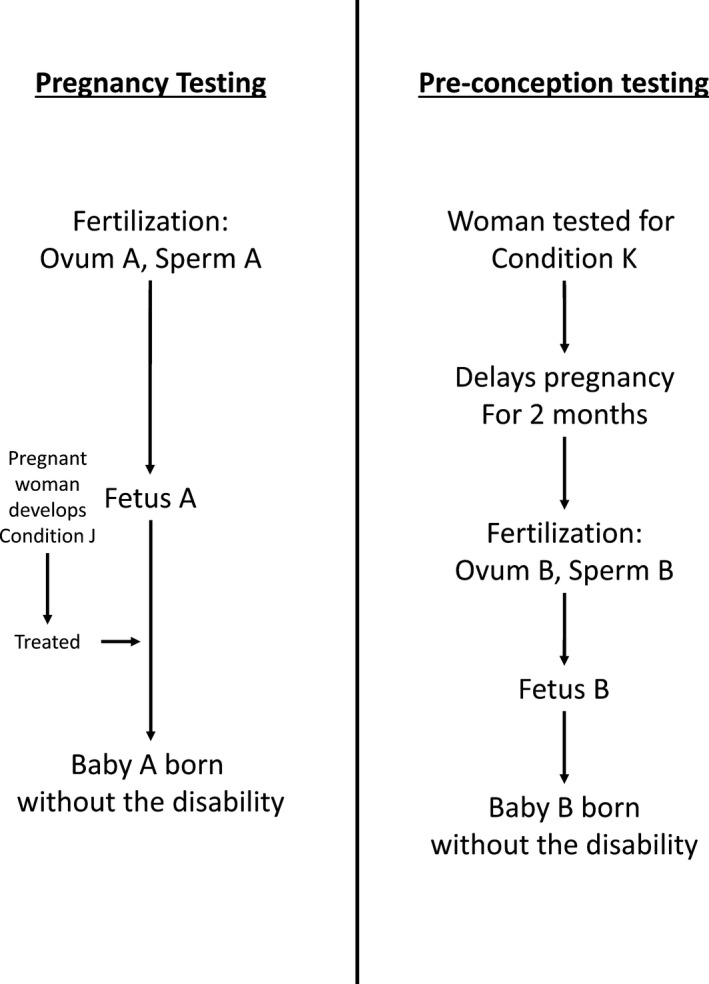
A diagram of Parfit's “Medical Programmes” thought experiment

The ethical implications of changing the identity of future people were famously explored by Parfit in his thought experiment, ‘The Medical Programmes.’^25^ He described two imaginary diseases, Condition J and Condition K, that will cause disability in a fetus if pregnant women are affected by the disease (the disability will reduce future quality of life, but life will still be worth living for the child). Condition J has a simple curative treatment, whereas Condition K has no treatment but resolves spontaneously in two months. Parfit imagined two hypothetical public health programmes: ‘Pregnancy testing’ would test millions of pregnant women for Condition J and treat them if necessary; on the other hand, ‘Pre‐conception testing’ would test for Condition K in millions of women who want to become pregnant, and advise those with the disease to delay conception for two months. Each programme would predictably avoid 1,000 cases of the disability, but in the thought experiment we only have enough money to fund one programme. Figure 1 outlines this thought experiment.

The two programmes are equivalent except that Pregnancy testing for Condition J is ‘person‐affecting’ (it benefits people who will exist in the future), whereas the benefit of Pre‐conception testing for Condition K is ‘impersonal’ (it changes which people will exist in the future for the better). If we do not fund Pregnancy testing for Condition J, 1,000 babies will be born disabled who could have been healthy if we had chosen differently. Those babies would be worse off because of our choice, and when they grow up they could blame us for harming them (or, at least, failing to make them better off). On the other hand, if we do not fund Pre‐conception testing for Condition K then 1,000 babies will still be born disabled, but since their lives will still be worth living they would be no worse off because of our choice; if we had chosen otherwise, they would never have existed at all. They could not blame us for our choice, and could not coherently claim that we harmed them (or failed to make them better off).

**Figure 2:**
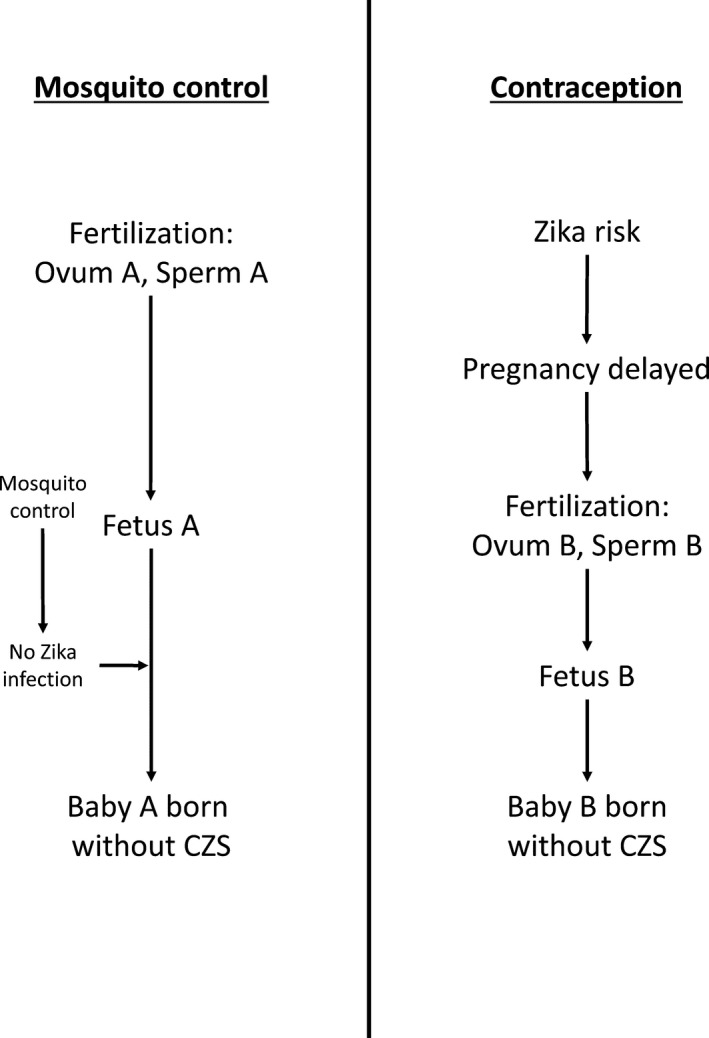
The identity implications of different interventions for Zika. Mosquito control offers a person affecting benefit, while the benefit of contraception is impersonal.

As illustrated in Figure 2, Parfit's thirty‐year‐old thought experiment presciently mirrors some of the choices posed by the Zika epidemic between prioritising the funding of person‐affecting interventions like mosquito control versus interventions with impersonal benefits like contraception. While in reality there is a not a strict requirement to choose one intervention over the other, we do have to choose how to allocate limited healthcare resources between different interventions and how much priority to give to each. This could be especially true in the case of Zika, since it has primarily affected poorer countries where limited health resources may lead to significant pressure to prioritise the best interventions.^26^

### 2.2 Different views of the non‐identity problem

There are different philosophical positions on these issues of the identity and wellbeing of future people. In his 1973 paper *Moral Problems of Population*,^27^ Jan Narveson famously defended a ‘person‐affecting view’ – that we should make people happy rather than making happy people. In his view, there are no positive duties to have children. (There could be duties to *not* have children if bringing more children into the world would infringe on the rights of others or if the quality of life of a child would be so low as to make its life not worth living.) He uses the example of two worlds: one has a small, hardy population that virtuously battles against the elements in a rugged landscape, and the other is full of bustling cities with thriving cultures. Would it be better for one or the other world to exist? Narveson claims that as long as the people of both worlds are similarly happy, then these two worlds are equally desirable. Likewise, Rebecca Bennett has argued that intuitive responses to scenarios like the one above are a matter of taste or preference, but not of morality.^28^ Because Narveson and Bennett think our policies should focus on improving the lives of existing people,^29^ it seems they would prioritise person‐affecting interventions like mosquito control. There would be no social‐justice‐based reason to use contraception to tackle Zika. (The person‐affecting view might support contraception if infants with CZS have such severe disability that their lives are not worth living. While this is plausible, at least in some of the more severe cases, it seems unlikely to apply to all cases of CZS).

However, others reject the person‐affecting view. Current policies affecting climate, natural resources or the environment may have profound impacts in several generations time, but they will not harm specific people because the interventions also change which people are born.^30^ Many people may feel that if our current energy policies would lead to future people living much less happy lives (or drastically reduce the Earth's population), then this would be profoundly wrong from the point of view of morality or social justice.

Another view holds that impersonal considerations, although they matter, are of secondary importance *vis‐a‐vis* person‐affecting concerns. For example, imagine that we had to choose between paying for a medical treatment that would cure cancer in a current 8‐year‐old child, or pre‐implantation genetic diagnosis that would mean a child will be born who will not develop cancer at age 8. In that case, it seems plausible that there are additional moral reasons to cure cancer in the current child. Those moral reasons potentially derive from our duties as parents, health professionals or society to the current child, and from deontological norms of beneficence and non‐maleficence. Although this case is arguably different from Zika since it deals with a person who already exists rather than a fetus (which is arguably not yet a person), some people could still accept what we could call a ‘Person‐affecting Priority view,’ that would mean giving greater priority to mosquito control over contraception. The practical impact of this view would depend on how *much* weight we give to person‐affecting reasons, as opposed to impersonal ones. If contraception were much more effective at preventing CZS, it may be that we should still fund it over mosquito control on the Person‐affecting Priority view. It would be important to know the relative weight of these different considerations as well as the relative effectiveness of different interventions.

Derek Parfit articulated and defended a different response to dilemmas like this. According to the ‘no‐difference view,’ person‐affecting and impersonal considerations are equally morally important. Parfit argued that what matters is the overall wellbeing of the people who will eventually exist. In the Medical Programmes thought experiment, he contended that since an equal number of cases of disability would be avoided by Pregnancy testing or Pre‐conception testing, intuitively there is no moral difference between them.^31^ The no‐difference view would imply a simpler solution for interventions to avoid CZS: since there is no moral difference between person‐affecting and impersonal considerations, the important thing would be to know which intervention is most cost‐effective.

Finally, one possibility is that CZS itself is seen as identity‐affecting. The full neurocognitive manifestations of CZS are not yet known, but it appears to cause profound neurological disability, at least in the most severe cases. Some philosophers like Jeff McMahan see identity as being determined by physical and psychological continuity rather than genetics.^32^ By causing significant changes to the nervous system before consciousness develops, and by radically changing the psychological capacities of the fetus, CZS might lead to the development of a child with no psychological continuity to the person they would have been without CZS. If this is the case, all methods to avoid CZS are potentially identity‐determining and impersonal. This would mean that we should focus our resources on the interventions which are most cost‐effective. However, it is likely that there is a spectrum of effect, with CZS sometimes being identity‐altering (in profound cases) and sometimes being identity‐preserving (in mild cases).

These different views of the non‐identity problem, some of their major supporters and the implications they would have on our response to Zika are outlined in Table 1 below.^33^^,^ 34 ^,^ 35 ^,^ 36 ^,^ 37 ^,^ 38

**Table 1:** Different views on non‐identity

Stance on non‐identity and Zika	Philosophers expressing support for view	Implications for social justice and public health response to Zika
Person‐affecting principle	Narveson Bennett	Fund Mosquito Control (no value placed on contraception)
Person‐affecting‐priority view	McMahan Savulescu Arrhenius	Mosquito control given relative priority over contraception if equally effective
No‐difference view	Parfit	Mosquito control and contraception are equally valued

### 2.3 How should our response to the non‐identity influence how we prioritise contraception?

#### 2.3.1 Only provide person‐affecting interventions

The person‐affecting principle would have some potentially unpalatable conclusions for social justice. For example, it would potentially mean that we would have no reason to preserve the environment for people who will exist in several generations’ time. We would also have to fund mosquito control instead of contraception even if it was thousands of times less cost‐effective in reducing the incidence of CZS. People who hold this view might be willing to bite this bullet, but for the purposes of this paper we will consider these implications too implausible and dismiss this view.

#### 2.3.2 Priority to person‐affecting interventions

As noted, another possibility is that impersonal benefits have some moral weight, but not as much as person‐affecting ones. If the Person‐affecting Priority view is correct, the moral difference between person‐affecting and impersonal benefits has to be weighed against other factors such as the cost‐effectiveness, practicality and associated consequences of different interventions. In the case of Zika, there are additional consequences that might favour contraception: it could have significant benefits for the vulnerable women most impacted by Zika (in a way that mosquito control would not), and it could alleviate poverty as well as advancing women's rights.

In Latin America and the Caribbean, 55% of pregnancies are unplanned, the highest rate of any region in the world.^39^ Up to 25% of women of reproductive age do not have access to contraception, and most of those who do use less reliable methods such as hormonal pills. Access to family planning methods is also closely linked to socioeconomic status: the poorest in society have the least access to contraception and the highest fertility rate (as well as the highest likelihood of being exposed to the Zika virus).^40^^,^ 41 Improving free access to more effective and long‐lasting contraceptives (such as hormonal intrauterine devices and subdermal implants) could significantly improve reproductive health outcomes and equality, as well as addressing the ongoing Zika outbreak.

Providing access to contraception could also improve women's health outcomes by reducing the rate of unsafe abortions in the region. Latin American countries with active Zika transmission have seen demand for abortion soar,^42^^,^ 43 despite limited or non‐existent access to legal abortion and the serious health risks of illegal and unsafe abortion.^44^^,^ 45 This will potentially add to the significant proportion (10%) of maternal deaths in the region caused by unsafe abortions.^46^ Providing contraception would therefore likely reduce maternal morbidity and mortality as well as potentially having psychological benefits for women.

A CDC study created a decision tree cost‐effectiveness model for Puerto Rico, assuming a year‐long Zika outbreak.^47^ It focused on 163,000 women who wanted to avoid becoming pregnant, and compared a control scenario with no intervention against a scenario of free same‐day provision of contraception and counselling. The model estimated that besides reducing the incidence of CZS by 25%, the US$33.5 million contraception programme would avoid US$170.7 million of future costs, for a total return on investment of 510%. There are no data on the cost‐effectiveness of mosquito control programmes to reduce the incidence of CZS, so it is unclear how cost‐effective contraception is compared to other available interventions. But until this becomes clear, we have evidence that contraception is not only cost‐effective but generates significant net savings.

Besides generating these savings for the health budget of affected countries, contraception could also directly target poverty by reducing the fertility rate: large families have to stretch their budget across more children, reducing the amount of food education and healthcare ‘invested’ in each child and reducing their future productivity.^48^ Moreover, it seems likely that even in poor countries with high fertility rates, women will only have a limited number of children. Contraception reduces the chance that one or more of those children will have CZS, which is better for families and society.

While contraception has impersonal benefits for future people, the above benefits to women and families are person‐affecting. If person‐affecting benefits carry more weight than impersonal benefits, all else being equal, then these reasons are even more compelling for providing access to contraception.

However, there are also potential (person‐affecting) drawbacks of contraception. The benefit of contraception would be through having women delay pregnancy until they are safe from Zika. It seems likely that once women consider themselves safe, or the authorities declare the Zika threat over, there will be a surge in women getting pregnant and a subsequent surge of children being born. Some have pointed out that this could put significant strain on the maternal health services of affected countries, as well as the education system once the children reach school age.^49^ The adverse outcomes from this spike in population might be significant but it is doubtful that they would outweigh the benefits contraception has in reducing the incidence of CZS. A partial amelioration of this problem would be to reduce the length of time women are advised to delay their pregnancy. Since the mosquitos that transmit Zika are seasonal, if women delay their pregnancy only for the duration of peak Zika transmission then the disruption to the birth rate would be minimised while still protecting a significant number of pregnancies.^50^ But given that human birth rates are normally seasonal (with the regional peak in August‐October)^51^ without any advice on the timing of pregnancies,^52^ it seems likely that using contraception to delay pregnancy will only alter a long‐standing pattern that does not put excessive strain on healthcare and education.

The effectiveness of providing access to contraception has also been questioned. There is some evidence from the Profamilia contraception programme in Colombia that improving access to contraception only causes a small reduction in the fertility rate in communities with low demand for contraception.^53^ But this may not apply to Zika, since the surge in demand for illegal abortions probably signals high concern to prevent CZS, and a desire to use contraception if available.^54^^,^ 55

#### 2.3.3 No difference

The third possibility noted above is that questions of identity make no moral difference in our prioritisation of contraception.

Parfit drew support for the no‐difference view from his own intuitions about the Medical Programmes thought experiment. Particularly when we think about population‐level interventions that could avoid many cases of illness in individuals not yet born, it seems unimportant whether these interventions would prevent illness in a fetus already conceived, or delay conception so that a different child is born. The two problems at the heart of the Zika outbreak are that it is causing thousands of babies to be born with profound disabilities, and that it is placing considerable psychological and financial stress on affected families – in particular poorer and younger women who are already some of the most vulnerable people in society.^56^ Both of these problems might be addressed equally well by contraception and mosquito control, at least if both are assumed to be equally cost‐effective. One interesting question, to which we will return shortly, is whether Parfit's no‐difference intuition is shared by the general population.

A different justification for this view is based on identifying what would be bad about failing to prevent cases of CZS. Faced with some of the puzzles arising from the non‐identity problem, some philosophers have argued that changing which people exist for the worse can wrong them, even if they are not ‘harmed.’ For example, some philosophers have argued that future people have rights to a sufficiently good start to life; they may be wronged by being born even if they have a life worth living.^57^ Along similar lines, Shiffrin has argued in favour of harm being non‐comparative (not requiring anyone to be made worse off),^58^ while Harman has contended that people can be harmed by being put into an undesirable state, even if they are not made worse off.^59^ On these views, if we failed to provide contraception and children were born with CZS, then they would have been (non‐counterfactually) harmed or wronged.

Finally, even if non‐identity matters morally, we may be tempted to regard contraception and mosquito control as equivalent, since both may be identity affecting. As noted above, that could be because of the profound effect of CZS itself on the identity of a future child. It could also result from the sensitivity of genetic identity to small changes in the timing of conception. It seems plausible that the extra time taken to wear insect repellent or to take a different road home to avoid an area with heavy insecticide spraying would change the genetic constitution of future people, since even the slightest change in the timing of conception could change which of the hundreds of millions of possible spermatozoa ends up fertilising the ovum. Almost any intervention to prevent CZS could slightly delay or hasten conception, and thus render its benefits impersonal. Of course, mosquito control may not change the identity of all the babies born shortly thereafter, though it could change the identity of some. If this is the case, then mosquito control would have a mix of impersonal and person‐affecting benefits; since there is no way of knowing how many people's identity will be affected, it would be impossible to weigh up these different effects. Although this does not apply to treatments for fetuses that are already infected, or interventions that prevent existing fetuses from being infected, it does apply to the majority of interventions we will use in the near future. This would mean that the non‐identity problem does not provide a tangible reason to prioritise other interventions over contraception in tackling Zika.

To summarise the analysis so far: we have looked at different views on the non‐identity problem, as well as different ways that it may influence our decision making about responses to Zika. We reject the claim that person‐affecting benefits are always better than impersonal ones, because that would lead to implausible conclusions. We argued that if even if person‐affecting benefits are somewhat better than impersonal ones, there may still be strong reasons to use contraception against CZS because of its person‐affecting benefits. Finally, we suggested that non‐identity might make no moral difference either if the no‐difference view is correct, if we accept non‐counterfactual accounts of harm, or if mosquito control or CZS itself are identity‐affecting.

## 3 A SURVEY OF THE PUBLIC'S INTUITIONS ABOUT THE NON‐IDENTITY PROBLEM

If philosophers are divided about the non‐identity problem and its implications for social justice, what do others think? Knowing the general public's intuitions about the non‐identity problem is potentially valuable. Firstly, it can reveal how willing they would be to make use of an impersonal intervention like contraception if it were made available. Secondly, the moral intuitions of the public can provide a counterbalance to philosophical reasoning; if philosophers reach a conclusion that is very different to the public's intuitions, that may lead to a re‐examination of the philosophical reasoning. Rawls called this general idea reflective equilibrium,^60^ arguing that analysis and intuitions should play an interactive role in determining our normative conclusions.^61^ To our knowledge, there are no published data on the views of the general public on the non‐identity problem, especially applied to a real‐world problem, or its implications for social justice. This is unfortunate given that the non‐identity problem is so often appealed to in discourse surrounding numerous bioethical topics.

With these reasons in mind, we conducted an online survey of a sample of the general public to measure their intuitions about the non‐identity problem. We hypothesised that:

1. Participants would be strongly influenced by the relative efficacy of interventions, and would prefer an intervention if it avoided more cases of CZS
2. A subset of participants would prefer person‐affecting benefits after the non‐identity problem had been explained
3. Participants’ answers would be consistent between questions relating to Zika and Parfit's 14‐year‐old girl thought experiment
4. Participants’ answers would correlate with their demographic factors and ideological views

### 3.1 Methods

Participants were recruited through Mechanical Turk (Amazon.com, California), an online platform for recruiting workers from around the world to complete human intelligence tasks such as answering surveys. (Past research indicates that Mechanical Turk is a high‐quality source of research participants for surveys of this kind.)^62^^,^ 63 Participants completed the survey through the Qualtrics platform (Provo, Utah). Informed consent was gained before participants began the survey, and participants were paid US$1 for a valid and complete response. Data from the survey were stored through Qualtrics, and analysed using IBM SPSS Statistics. We performed paired sample *t*‐tests to compare responses to questions, and assessed associations between participants’ responses and their demographic factors using correlation analysis. In both *t*‐tests and the correlations, a *p*‐value of <0.05 was considered statistically significant. We determined the number of participants needed using Creative Research System's Sample Size Calculator (http://www.surveysystem.com/sscalc.htm). We aimed to recruit 106 participants in order to obtain an 95% confidence interval of 10%, assuming a very large population and a 90% completion rate.

**Figure 3:**
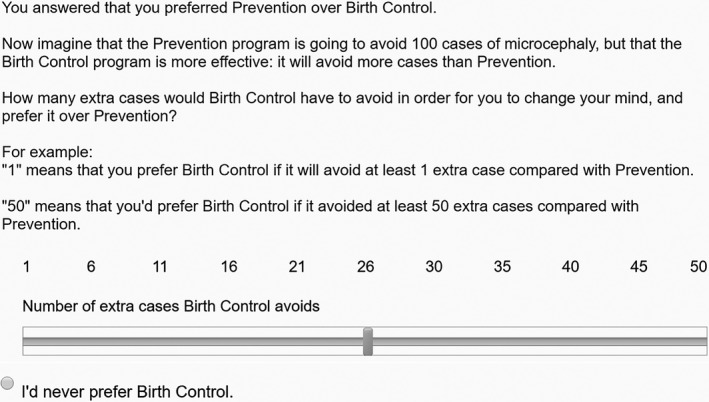
Example of the survey's willingness‐to‐pay style questions

The survey asked whether participants preferred (impersonal) contraception or (person‐affecting) mosquito control as interventions against CZS. In the survey, mosquito control was labelled ‘Prevention’ and contraception was labelled ‘Birth Control.’ The strength of their preference was first measured with a 7‐point Likert scale where 4 indicates “no preference” and 1 indicates “strongly prefer Birth Control” and 7 indicates “strongly prefer Prevention”, meaning that responses below 4 represent an overall preference for mosquito control and responses above 4 represent an overall preference for contraception. Next, their preference was measured with a version of a ‘willingness‐to‐pay’ question, where participants were asked how many more cases of CZS would need to be avoided in order for them to change their preference (from 1‐50 extra cases). These questions measured the strength of respondents’ relative preference. Figure 3 above shows the willingness‐to‐pay style question participants would be asked if they previously preferred mosquito control over contraception.

**Figure 4:**
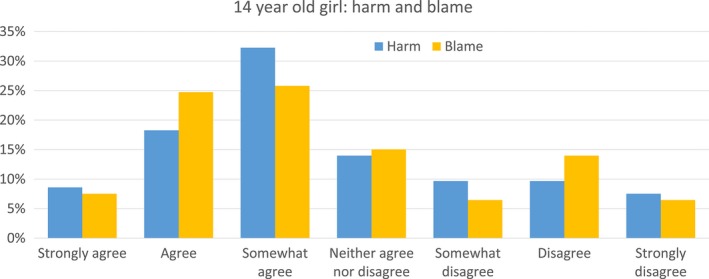
Participants’ answers to the questions of whether the 14‐year‐old girl choice harmed her child, and whether the child could blame her for her choice [Colour figure can be viewed at wileyonlinelibrary.com]

**Figure 5:**
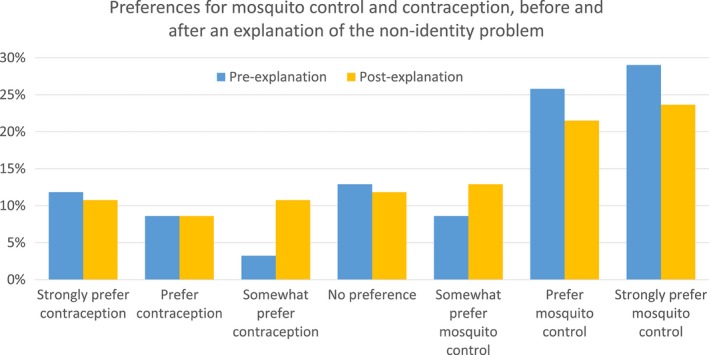
Participants’ answers on whether they prefer mosquito control or contraception as a way to tackle CZS. Measured before and after the non‐identity problem was explained [Colour figure can be viewed at wileyonlinelibrary.com]

The survey first asked questions about person‐affecting and impersonal interventions to tackle Zika, both before and after explaining non‐identity problem, to determine whether familiarity with (and, hopefully, understanding of) the problem changed participants’ preferences. The explanation of the non‐identity problem is included in Appendix 1. The survey went on to measure participant's intuitions about Parfit's ‘14‐year‐old girl’ thought experiment (Box 1) using 7‐point Likert scales.

**Box 1: 14‐year‐old girl thought experiment, as described in our survey. Wording adapted from Parfit^64^**

Imagine that there is a 14‐year‐old girl who chooses to have a child.

Because she is so young, it is likely that her child (“James”) will have a bad start in life. Though this will have bad effects throughout James’ life, his life will still be worth living.

If this girl had waited for several years, she would have had a different child (“Jane”), who would be likely to have a better start in life.

The survey concluded with a demographics section measuring age, gender, nationality, education, income, number of children, political ideology (including items like liberalism and conservatism), religiosity (including the morality of contraception and abortion), and utilitarian tendency (Oxford Utilitarianism Scale).^65^ A full version of the survey can be found in Appendix 1. The Oxford University Central University Research Ethics Committee and the Monash University Human Research ethics Committee gave the survey ethics approval on the 31st of May and 1st of June 2016, respectively (see Appendix 2). The full survey included items measuring preferences for other interventions besides mosquito control and contraception, but since these are not relevant to the non‐identity problem, which is the focus of this article, we do not report them here.

### 3.2 Results

We recruited 108 participants, of whom 15 gave incomplete responses which were excluded, leaving 93 valid responses. The vast majority identified themselves as from the US (96%), while they had a wide range of ages from 19‐71, with a mean of 37 (SD = 12.6). Just over half the responses (54%) were from women. The majority (83%) had at least attended college, and 53% had a bachelor's degree or higher. Just under half the cohort (48%) had children.

Before we explained the non‐identity problem, participants on average were more likely to favour mosquito control to prevent cases of CZS than contraception, even when it was specified that these would avoid the same number of cases of (*M* = 4.91, *SD* = 2.09). Explaining the non‐identity problem led to no significant change in preference for either intervention, *t*(92) = 1.504, *p* = .14.

Before the explanation of the non‐identity problem, a minority of participants (13%) answered that they had no preference, and a larger proportion (28%) of participants would change their preference if the alternative intervention would avoid 10 or fewer additional cases of microcephaly. On the other hand, a sizeable minority of participants would only change their preference from mosquito control to contraception if it would avoid 46‐50 more cases of microcephaly (14%), or would never change their preference regardless of effectiveness (17%).

Participants scoring higher on the Oxford Utilitarianism Scale were more likely to have a higher preference for contraception than mosquito control, both before the non‐identity problem was explained (*r* = ‐.30, *p* = .004) and after (*r* = ‐.32, *p* = .002).

When asked specifically about Parfit's thought experiment, participants felt on average that the girl's choice to have a child so young was wrong (*M* = 3.24, *SD* = 2.04), they felt that the girl's choice to have a child would harm her child (*M* = 2.95, *SD* = 2.00), and that the child could blame her for her choice (*M* = 4.87, *SD* = 2.02). There was a significant correlation between participants preferring mosquito control over contraception (after the non‐identity problem was explained) and answering that the 14‐year‐old girl's choice was wrong (*r* = .21, *p* = .04), but there was no significant correlation between mosquito control preference and judgments that the girl harmed her child (*r* = .08, *p* = 0.47) nor judgments that the child could blame her (*r* = .09, *p* = .39).

Participants’ responses were not significantly associated with gender, income, or political ideology.

### 3.3 Discussion

This is the first survey to assess views about the non‐identity problem in relation to public health and social justice. We assessed preferences for different types of public health intervention to avoid cases of CZS, as well as directly asking participants about their views on a classic philosophical thought experiment relating to the non‐identity problem.

Before the non‐identity problem was explained, the majority of participants preferred mosquito control over contraception as a way to control CZS. There are several possible reasons for this. It may indicate an intuitive preference for person‐affecting interventions, or alternatively that participants may prefer interventions that do not impinge on people's reproductive freedoms and disrupt their plans for pregnancy. It may also be that people see mosquito control as dealing with the root problem of Zika, rather than merely accommodating the problem (as more of a ‘symptomatic’ treatment). They may also have factored in the possible indirect health benefits of mosquito control, like reducing the transmission of other mosquito‐borne diseases.

The explanation of the non‐identity problem did not significantly affect preferences. We had hypothesised that respondents would be more likely to choose a person‐affecting intervention after explanation of the non‐identity problem (which we would not assume most lay people appreciate without explanation), but in fact there was a small (non‐significant) shift in responses towards contraception. It may be that participants did not understand the explanation of the non‐identity problem.

A large proportion of participants (41%) either had no preference between Zika interventions, or would change their preference to avoid only a few additional cases of microcephaly. Some of these respondents presumably support the no‐difference view. For the 22% of participants who preferred mosquito control but would change their preference to contraception if it avoided only a few more cases (<10), it appears that even if they preferred a person‐affecting benefit, this preference could be outweighed by small difference in effectiveness between the two interventions. This might show that these participants endorse a version of what we have labelled the ‘Person‐affecting Priority’ view: other things being equal they prefer person‐affecting benefits over impersonal ones, but they do not place much weight on the difference.

A reasonable number of participants (31%) would only change their preference from mosquito control to contraception if it would avoid many more cases of microcephaly (46‐50 or never), though this (latter) number dropped to 11% once the non‐identity problem was explained. This could reflect support for the person‐affecting principle, or maybe an aversion to contraception as a public health intervention, potentially for reasons suggested above. This response was not associated with religious views. (Future surveys could replace contraception with an intervention that just advised women to delay their pregnancy. This would remove the effect that any moral qualms with contraception might have on participants’ answers).

In response to the Parfit thought experiment, most participants thought the 14‐year‐old girl's choice to become pregnant was wrong. It may be that they intuitively subscribe to something like Parfit's no‐difference view, or it may be that they have qualms with teenage pregnancy regardless of the non‐identity problem. Curiously, this response was weakly correlated with respondents preferring mosquito control, which would not be compatible with the no‐difference view.

Interestingly, most participants agreed with the statements that the girl had harmed her child and that the child could blame her. This is in spite of the fact that the earlier explanation of the non‐identity problem implied that this child would not otherwise have existed. These counterintuitive findings suggest that either most participants did not understand the non‐identity problem, or hold a non‐counterfactual view about harm and blame that can still apply in non‐identity cases, similar to that of Harman and Shiffrin.^66^

Looking at these answers together, participants either did not understand the non‐identity problem or did not see it as very morally important for decision‐making about public health interventions. The survey was limited by its small sample of the US general public, and it would be valuable to know whether these findings are replicated in other populations. We also were not able to explore the reasons behind responses, and future qualitative research may help uncover those. Moreover, opinion polls are not necessarily an accurate reflection of participants’ true beliefs, and responses may be influenced by what participants think their answers ought to be. However, these preliminary findings suggest that (other things being equal) the general public has a weak preference for mosquito control over contraception as a public health intervention, though this was not obviously related to the non‐identity problem.

## 4 CONCLUSION

The ongoing Zika outbreak is a serious public health problem affecting many people around the world. Contraception may be a powerful tool for reducing the cases of CZS, but it raises significant philosophical, ethical and social justice questions – particularly since it confers its benefits by changing which people will exist in the future. This paper aimed to shed light on the relevance of the non‐identity problem for Zika through both ethical analysis and an empirical survey.

In the ethical analysis, we argued that non‐identity should not significantly affect our prioritisation of contraception in tackling CZS. There is some reason to think that there is no moral difference between person‐affecting and impersonal benefits, and in any case most CZS interventions are likely to be impersonal to at least some degree since they influence the timing of conception. Furthermore, CZS itself might be identity‐affecting, at least in some cases. We also argued that even if non‐identity does give us some reason to prefer person‐affecting benefits over impersonal ones, this reason needs to be weighed against the indirect benefits of using contraception to tackle CZS, many of which are person‐affecting themselves.

The empirical survey provided a preliminary exploration of the general public's attitudes. It suggested that most people would not see the non‐identity problem as a reason to reject contraception as a way to tackle CZS. Although many preferred mosquito control over contraception as a public health intervention, the largest group (41%) would prefer to fund the most effective intervention to avoid CZS. Moreover, it is likely that if the public prefer mosquito control over contraception, it is for reasons other than the non‐identity problem.

Although the non‐identity problem is philosophically important, can affect questions of social justice and may have significant implications for other policy questions, we conclude that non‐identity should not affect our response to CZS. Improving access to contraception is at least as deserving of funding as other public health measures, such as mosquito control and research into Zika. This conclusion can apply not only to Zika, but to other epidemics of teratogenic diseases where contraception could be useful. Epidemics of these teratogenic diseases have profound effects on children and families. Interventions to avoid potentially devastating disabilities should be prioritised on the basis of their effectiveness, cost and practicality, not on whether they are identity‐affecting. Our empirical survey shows there is the potential that the public would prefer interventions like mosquito control over contraception, and careful thought needs to be given to public concerns before rolling out any programme involving widespread promotion of contraception as a mode of dealing with an infectious disease outbreak.

## FUNDING

Julian Savulescu was supported by Wellcome Trust grant WT 104848/Z/14/Z and by the Oxford Martin Programme on Collective Responsibility for Infectious Disease.

Dominic Wilkinson was supported for this work by a grant from the Wellcome Trust WT106587/Z/14/.

## CONFLICT OF INTEREST

No conflicts Declared.

## Notes

1 World Health Organization. (2016). *WHO Statement on the First Meeting of the International Health Regulations (IHR 2005) Emergency Committee on Zika Virus and Observed Increase in Neurological Disorders and Neonatal Malformations*. Available from: http://www.who.int/mediacentre/news/statements/2016/1st-emergency-committee-zika/en/ [Accessed 22 July 2016].

2 Honein MA, et al. (2016). Birth Defects among Fetuses and Infants of US Women with Evidence of Possible Zika Virus Infection During Pregnancy. *JAMA. 317*(1), 59‐68.

3 Rasmussen SA, et al. (2016). Zika Virus and Birth Defects — Reviewing the Evidence for Causality. *New England Journal of Medicine*. (374), 1981‐1987.

4 Thurn J. (1988). Human Parvovirus B19: Historical and Clinical Review. *Reviews of Infectious Diseases. 10*(5), 1005‐11.

5 Guo XX, et al. (2016). Culex Pipiens Quinquefasciatus: A Potential Vector to Transmit Zika Virus. *Emerging Microbes and Infections. 5*, e102.

6 Marcondes CB, Ximenes MFFM. (2016). Zika Virus in Brazil and the Danger of Infestation by Aedes (Stegomyia) Mosquitoes. *Revista da Sociedade Brasileira de Medicina Tropical. 49*(10), 4‐10.

7 Harris LH, Silverman NS, Marshall MF. (2016). The Paradigm of the Paradox: Women, Pregnant Women, and the Unequal Burdens of the Zika Virus Pandemic. *The American Journal of Bioethics. 16*(5), 1‐4.

8 British Broadcasting Corporation (2014). *Moral Case against Contraception*. Available from: http://www.bbc.co.uk/ethics/contraception/against_1.shtml [Accessed 5 January 2017].

9 Baeten JM, et al. (2001). Hormonal Contraception and Risk of Sexually Transmitted Disease Acquisition: Results from a Prospective Study. *American Journal of Obstetrics and Gynecology. 185*(2), 380‐385.

10 Chong A, et al. (2013). Effectiveness and Spillovers of Online Sex Education: Evidence from a Randomized Evaluation in Colombian Public Schools. *National Bureau of Economic Research*.

11 Fennell JL. (2011). Men Bring Condoms, Women take Pills: Men's and Women's Roles in Contraceptive Decision Making. *Gender and Society. 25*(4), 496‐521.

12 Beck J. (1 July 2016). The Importance of Contraception to the Zika Fight. *The Atlantic*.

13 Parfit D. (1984). *Reasons and Persons*. New York, NY: Oxford University Press.

14 Hope T, McMillan J. (2012). Physicians’ Duties and the Non‐Identity Problem. *The American Journal of Bioethics. 12*(8), 21‐29.

15 Narveson J. (1973). Moral Problems of Population. *The Monist. 57*(1), 62‐86.

16 Hope, McMillan, *op. cit*. note 14, p. 5.

17 Sher G. (2005). Transgenerational Compensation. *Philosophy & Public Affairs. 33*(2), 181‐200.

18 Bowie WR, King AS, Werker DH, et al. Outbreak of Toxoplasmosis Associated with Municipal Drinking Water. *The Lancet. 350*(9072), 173‐177.

19 Jensen IP, Thorsen P, Jeune B, et al. (2000). An Epidemic of Parvovirus B19 in a Population of 3596 Pregnant Women: A Study of Sociodemographic and Medical Risk Factors. *BJOG: An International Journal of Obstetrics & Gynaecology. 107*(5), 637‐643.

20 Ujiie M, Nabae K, Shobayashi T. Rubella Outbreak in Japan. *The Lancet. 383*(9927), 1460‐1461.

21 Elfiky AA. (2016). Zika Viral Polymerase Inhibition Using Anti‐HCV Drugs Both in Market and under Clinical Trials. *Journal of Medical Virology. 88*(12), 2044‐2051.

22 Dyer O. (2016). Zika Vaccine Could Be in Production by Year's End, Says Maker. *BMJ. 352*(i630).

23 Martinez‐Bakker ME. (2016). Preventing Zika Virus Infection during Pregnancy Using a Seasonal Window of Opportunity for Conception. *PLoS Biology. 14*(7), e1002520.

24 World Health Organization. (2016). *Zika Strategic Response Framework & Joint Operations Plan*. Available from: http://www.who.int/emergencies/zika-virus/strategic-response-framework.pdf [Accessed 22 July 2016].

25 Parfit, *op. cit*. note 10, p. 2.

26 Centre for Disease Control and Prevention. (2016). *All Countries and Territories with Active Zika Virus Transmisison*. Available from http://www.cdc.gov/zika/geo/active-countries.html [Accessed 2 April 2016].

27 Narveson, *op. cit*. note 17, p. 4.

28 Bennett R. (2009). The Fallacy of the Principle of Procreative Beneficence. *Bioethics. 23*(5), 265‐273.

29 Narveson, *op. cit*. note 17, p. 4.

30 Parfit, *op. cit*. note 10, p. 2.

31 Ibid.

32 McMahan J. Wrongful Life: Paradoxes in the Morality of Causing People to Exist. In: Coleman JL, Morris CW, editors. *Rational commitment and social justice: Essays for Gregory Kavka*. Cambridge, UK: Cambridge University Press, 1998; p. 208‐249.

33 Arrhenius G. (2005). The Person‐Affecting Restriction, Comparativism, and the Moral Status of Potential People. *Ethical Perspectives. 10*(3), 185‐195.

34 Bennett, *op. cit*. note 18, p. 4.

35 Coleman, Morris, *op. cit*. note 22, p. 5.

36 Narveson, *op. cit*. note 17, p. 4.

37 Parfit, *op. cit*. note 10, p. 2.

38 Savulescu J. (2001). Procreative Beneficence: Why We Should Select the Best Children. *Bioethics. 15*(5‐6), 413‐426.

39 Bahamondes L, Ali M, Monteiro I, et al. (2017). Contraceptive Sales in the Setting of the Zika Virus Epidemic. *Human Reproduction. 32*(1), 88‐93.

40 Diniz D, Gumieri S, Bevilacqua BG, et al. (2017). Zika Virus Infection in Brazil and Human Rights Obligations. *International Journal of Gynecology & Obstetrics. 136*(1), 105‐110.

41 Langer A, Caglia JM, Menéndez C. (2016). Sexual and Reproductive Health and Rights in the Time of Zika in Latin America and the Caribbean. *Studies in Family Planning. 47*(2), 179‐181.

42 Aiken AR, et al. (2016). Requests for Abortion in Latin America Related to Concern About Zika Virus Exposure. *New England Journal of Medicine*. (375), 396‐398.

43 Miller ME. (20 February 2016) Zika Virus: Pregnant Women ‘Begging Online for Abortion Pills’ in Countries Where Terminations Are Illegal. *The Independent*.

44 Costa SH, Vessey MP. (1993). Misoprostol and Illegal Abortion in Rio De Janeiro, Brazil. *The Lancet. 341*(8855), 1258‐1261.

45 Faundes A, et al. (1996). Post‐Abortion Complications after Interruption of Pregnancy with Misoprostol. *Advances in Contraception. 12*(1), 1‐9.

46 Guttmacher Institute. (2016). *Fact Sheet ‐ Abortion in Latin America and the Caribbean*. Available from: https://www.guttmacher.org/sites/default/files/factsheet/ib_aww-latin-america.pdf [Accessed 14 July 2016].

47 Li R, et al. (2017). Cost‐Effectiveness of Increasing Access to Contraception During the Zika Virus Outbreak, Puerto Rico, 2016. *Emerging Infectious Diseases. 23*(1), 74‐82.

48 Birdsall NM, Griffin CC. (1988). Fertility and Poverty in Developing Countries. *Journal of Policy Modeling. 10*(1), 29‐55.

49 Beard S. (20 February 2016). Using Birth Control to Combat Zika Virus Could Affect Future Generations. *The Conversation*.

50 Martinez‐Bakker, *op. cit*. note 23 p. 6.

51 Ibid.

52 Roenneberg T, Aschof J. (1990). Annual Rhythm of Human Reproduction: I. Biology, Sociology, or Both? *Journal of Biological Rhythms. 5*(3), 195‐216.

53 Miller G. (2010). Contraception as Development? New Evidence from Family Planning in Colombia. *The Economic Journal. 120*(545), 709‐736.

54 Aiken et al., *op. cit*. note 29, p. 6.

55 Miller, *op. cit*. note 30 p. 6.

56 Diniz et al., *op. cit*. note 36 p. 10.

57 Velleman JD. (2008). Persons in Prospect. *Philosophy & Public Affairs. 36*(3), 221‐244.

58 Shiffrin SV. (1999). Wrongful Life, Procreative Responsibility, and the Significance of Harm. *Legal Theory. 5*(2), 117‐148.

59 Harman E. Harm as Causing Harm. In: Roberts MA, Wasserman DT, editors. *Harming Future Persons: Ethics, Genetics and the Nonidentity Problem*. Dordrecht, Netherlands: Springer, 2009; p. 137‐154.

60 Rawls J. (1971). *A Theory of Justice*. Cambridge, Massachusetts: Harvard University Press.

61 Ibid.

62 Buhrmester M, Kwang T, Gosling SD. (2011). Amazon's Mechanical Turk: A New Source of Inexpensive, yet High‐Quality, Data? *Perspectives on Psychological Science. 6*(1), 3‐5.

63 Mason W, Suri S. (2012). Conducting Behavioral Research on Amazon's Mechanical Turk. *Behavior Research Methods. 44*(1), 1‐23.

64 Parfit D. (1984). *Reasons and Persons*. New York, NY: Oxford University Press, p. 358.

65 Kahane G, et al. (2016). Beyond Sacrificial Harm: Positive and Negative Dimensions of Everyday Utilitarian Decision‐Making. *Manuscript submitted for publication*.

66 Roberts, *op. cit*. note 59 p. 13.

## APPENDIX 1 – FULL SURVEY OF THE GENERAL PUBLIC'S INTUITIONS ON THE NON‐IDENTITY PROBLEM

### 1.1

Note: the questions from the Oxford Utilitarianism scale have been removed from this appendix, since the paper it is from is still under consideration.^67^

